# Development of IgY-Based Sandwich ELISA as a Robust Tool for Rapid Detection and Discrimination of Toxigenic *Vibrio cholerae*

**DOI:** 10.1155/2018/4032531

**Published:** 2018-10-02

**Authors:** Mahdiye Bayat, Alireza Khabiri, Behzad Hemati

**Affiliations:** ^1^Department of Microbiology, Islamic Azad University-Karaj Branch, Karaj 3148635731, Iran; ^2^Diagnostic Biotechnology Unit, Pasteur Institute of Iran, Research and Production Complex, Karaj, Iran

## Abstract

**Background:**

The conventional methods for diagnosis of *Vibrio cholerae* are time consuming, complicated, and expensive. Development of rapid detection tests is critical for prevention and management of cholera. This study aimed to introduce two sensitive sandwich ELISAs based on avian antibodies (IgY) targeting outer membrane protein W (OmpW) and cytotoxin B (CtxB) antigens of *V. cholerae*.

**Methods:**

The sequences of *ompW* and *ctxB* genes were cloned into pET28a vector. *Escherichia coli* BL21 (DE3) was transformed with the recombinant vectors, and gene expression was induced by IPTG. The expressed proteins were purified by affinity chromatography using Ni-NTA resins. Two groups of white Leghorn chickens were immunized by recombinant proteins, and the generated antibodies were purified from egg yolks of chickens by PEG precipitation. The antibodies were used for the development of *α*-OmpW and *α*-CtxB ELISAs.

**Results:**

The expression and purification yielded 59 and 38 mg of recombinant OmpW and CtxB, respectively, per one liter of bacterial culture. PEG precipitation and purification of egg yolk antibodies yielded on average (±SD) 66.5 ± 1.80 and 50.9 ± 2.23 mg of purified *α*-OmpW and *α*-CtxB per egg, respectively. The analytical sensitivity of *α*-OmpW ELISA was 103 cfu/mL of *V. cholerae* and that of *α*-CtxB ELISA was 33 pg/mL of recombinant cytotoxin B. The two developed ELISAs did not show any cross-reactivity to any tested bacteria grown in common conditions.

**Discussion:**

The current study is the first report on using IgY for detection of *V. cholerae*. The developed ELISAs were shown to have considerable analytical sensitivity and specificity. Therefore, the assays can be one of the convenient methods for sensitive and specific detection of toxigenic *V. cholerae* strains in clinical and environmental samples.

## 1. Introduction

Cholera is an important life-threatening diarrheal disease, transmitted through contaminated water and food. The causative agent of this disease is *Vibrio cholerae*, which emerged from Southwest Asia. There are around 200 different serogroups of *V. cholerae*, among which O1 and O139 are toxigenic [1]. Based on A, B, and C antigens in lipopolysaccharide, the O1 is classified into three different serotypes, Inaba, Ogawa, and Hikojima. In addition, based on some biological and biochemical properties, O1 is classified into two biotypes of Classic and El Tor [2].

The conventional diagnostic methods for detection of *V. cholerae* include the isolation and characterization of the causative agent. These laboratory tests are time consuming, labor-intensive, and expensive. On the other hand, lack of required facilities in small laboratories in remote areas makes the detection of organism difficult. In addition, there are some molecular methods for the detection of toxigenic *V. cholerae*, with high sensitivity and specificity [3, 4]. However, these methods have two fundamental problems: (1) they are expensive and require laboratory facilities and trained personnel and (2) presence of a gene coding exotoxin does not necessarily prove the production and secretion of the relevant protein. Hence, as the severity and rapid spread of the disease make it a serious potential problem, rapid, precise, and on time detection of toxigenic strains in clinical and environmental samples provide an important tool to monitor, control, and prevent the spread of the disease. Therefore, efforts have been made to develop rapid and simple diagnostic methods with acceptable sensitivity and specificity [5]. Among the rapid diagnostic methods, co-agglutination, ELISA, and dipstick ELISAs are the most important ones used in detection of *V. cholerae* in clinical and environmental samples [6–9].

As the sequence of outer membrane protein W (*ompW*) gene is conserved among different serogroups of *V. cholerae*, species-specific identification of the organism is possible by targeting OmpW antigens in immunoassays [10]. In addition, the strains harboring toxin genes (*ctxAB*) are the only producers of the toxin and are known as toxigenic [2]. Therefore, the presence of OmpW and CtxB antigens indicates toxigenic strains of *V. cholerae*.

In the present study, the IgY technology has been employed for the production of specific antibodies against OmpW and CtxB antigens of *V. cholerae*. The produced antibodies were used for development of two sandwich enzyme-linked immunosorbent assays (*α*-OmpW ELISA and *α*-CtxB ELISA) for the sensitive and specific detection of toxigenic *Vibrio cholerae* strains, for direct use in clinical or environmental samples.

## 2. Materials and Methods

### 2.1. Bacterial Strains

The bacterial strains used in this study were obtained from the microbial collection of Pasteur Institute of Iran and Culture Collection for Research and Industrial Microorganisms of Iranian Research Organization for Science and Technology (IROST).

The bacterial strains used for the evaluation of the specificity of the developed assays are listed in Table 1. Some of these bacteria are part of gastrointestinal (GI) microbiota and some are pathogenic agents of GI. As probability of the presence of the selected organisms in human fecal is high, they can be considered as potential source of cross-reactivity; therefore, they are used for evaluation of the specificity of the developed assays.

### 2.2. Polymerase Chain Reaction for Amplification of *ompW* and *ctxB* Genes

Genomic DNA from *Vibrio cholerae* O1 strain (Inaba) (PTCC No.: 1611) was extracted using GF-1 Bacterial DNA Extraction Kit (Vivantis, Malaysia, HQ). Based on published sequences (ompW sequence GenBank accession no. X51948 and cholera toxin B encoding *ctxB* gene sequence GenBank accession no. X00171), primer sets for the amplification of *ompW* and *ctxB* genes were designed (Table 2). PCR amplification of the target DNA was carried out with a reaction volume of 50 *µ*l. Each reaction mixture contained 2 *µ*l of template DNA (10 *µ*g/mL), 2 *µ*l of each primer (10 *µ*M), and 44 *µ*l of nuclease-free water in AccuPower® *Pfu* PCR PreMix tubes (Bioneer, Korea). The reaction mixture was subjected to 30 cycles of denaturation at 94°C for 30 s, annealing at 60°C and 55°C (for *ompW* and *ctxB*, respectively) for 30 s, and extension at 72°C for 45 and 30 s (for *ompW* and *ctxB*, respectively). Before initiation of the first cycle, the reaction mixture was heated at 94°C for 5 min to allow complete denaturation of the template and after the last cycle, the reaction mixture was heated at 72°C for 10 min to allow complete extension of the PCR products. The PCR products were evaluated on 1% agarose gel.

### 2.3. Cloning, Expression, and Purification of Recombinant OmpW and CtxB Proteins

The purified PCR product of *ompW* and *ctxB* genes were digested by *Bam*HI and *Xho*I restriction enzymes and directionally cloned into *Bam*HI- and *Xho*I-digested pET-28a, and the constructs were transformed to CaCl_2_-treated BL21 (DE3) strain of *Escherichia coli.* Transformants were selected and confirmed by the restriction digestion of their plasmids, as well as by colony PCR by the specific primers of each gene. The positive clones were induced to express recombinant proteins by a 4 h induction with 0.5 mM IPTG. The induced proteins were evaluated by SDS-PAGE analysis. The His-tagged recombinant proteins were purified by Ni-NTA chromatography according to the manufacturer's protocol (Qiagen, Germany). The concentration of purified proteins was determined by the method of Bradford [11].

### 2.4. Immunization of Chickens

Six 25-week-old white Leghorn chickens were obtained from the laboratory animal production center of the Pasteur Institute of Iran (Alborz, Karaj, Iran). The institutional and national guide for the care and use of laboratory animals was followed. The chickens were divided into three different groups. Immunization of the first and the second group was performed by intramuscular injection of 500 *µ*l of 10 *µ*g/mL of recombinant OmpW and CtxB, respectively, formulated with equal volume of complete Freund's adjuvant administrated into two sides of the chest area. Two subsequent boosters containing each recombinant protein emulsified with an equal volume of incomplete Freund's adjuvant and pure recombinant proteins were given on days 30 and 60, respectively. The last group (control group) was administered by PBS (pH 7.2) intramuscularly. One week after the last immunization, the eggs were collected daily, for two months, and stored at 4°C.

### 2.5. Purification of *α*-OmpW and *α*-CtxB Antibodies from Egg Yolks of Immunized Chickens

IgY antibodies were extracted from egg yolks using Pauly et al. method [12]. Briefly, twice the volume of yolk suspensions were mixed with PBS and then 3.5% (w/v) PEG 6000 (Merck, Germany) were added, and the suspension was shaken for 10 min at room temperature. Two different phases were separated by 20 min centrifugation at 13,000×*g* at 4°C. To remove the lipids, the collected supernatant was filtered by Whatman filter paper and 8% PEG 6000 was added to the filtrate. After shaking and centrifugation as described above, the pellet containing IgY was dissolved in 1 mL of PBS and the final volume was made to 10 mL by PBS. Then, 12% PEG 6000 was added to the solution, shaken by rotation, and centrifuged as described above. The pellet was resuspended carefully in 800 *µ*L PBS. The final extract was dialyzed overnight against normal saline with 3 exchanges. The concentration of the purified antibodies was evaluated by Bradford assay [11].

### 2.6. Evaluating the Reactivity of the Purified Antibodies

The specific activity of purified antibodies (*α*-OmpW and *α*-CtxB) was evaluated by Western blotting and indirect ELISA.

#### 2.6.1. Western Blotting

The purified OmpW and CtxB proteins were run on 12% SDS-PAGE gel and then electrotransferred to nitrocellulose membranes. The membranes were blocked with 1% bovine serum albumin (BSA) in PBS, overnight at 4°C. After washing twice with PBS buffer containing 0.1% Tween20 (PBS-T), each membrane was incubated with the corresponding purified antibody (*α*-OmpW or *α*-CtxB) at a dilution of 1 : 3,000 for 1 hour at room temperature. After extensive washing, the membranes were incubated with goat anti-chicken IgY-HRP (Abcam-UK) at a dilution of 1 : 4,000 for 1 hour and, after washing, the blots were developed by DAB and H_2_O_2_.

#### 2.6.2. Indirect Enzyme-Linked Immunosorbent Assay

The antibodies, purified from the collected eggs at the end of each week after immunization, were tested by indirect ELISA (16 eggs for each immunization group: *α*-OmpW, *α*-CtxB, and control). For this aim, a solution of purified antibodies (1 mg/ml) were diluted to 1 : 10,000 and 100 *µ*l of the diluted antibodies was incubated for 1 hour at 37°C, in microtiter plates coated with 2 *µ*g/ml of recombinant OmpW or CtxB. After washing thrice with PBS-T, the wells were incubated with 100 *µ*l of IgY goat anti-chicken IgY-HRP (Abcam-UK) at a dilution of 1 : 30,000 for 1 hour at 37°C. Subsequently, the plates were washed and the colorimetric reaction was carried out using 100 *µ*l of tetramethylbenzidine (TMB). The enzymatic reaction was stopped by adding 50 *µ*l of 1 M HCl, and the absorbance of each well was measured at 450 nm.

### 2.7. *α*-OmpW ELISA for Detection of *V. cholerae*

In order to optimize *α*-OmpW ELISA, bacterial suspensions of *V. cholerae* (PTCC No.: 1611) and *E. coli* (PTCC No.: 1395) with the density equivalent to 1 McFarland standard were used as positive and negative controls, respectively. A part of the dialyzed *α*-OmpW antibodies was diluted to 10, 5, 2.5, 1.25, 0.6, 0.3, and 0.15 *μ*g/mL in coating buffer (100 mM sodium carbonate-bicarbonate buffer with pH 9.6). Another part of dialyzed *α*-OmpW antibodies was conjugated with horseradish peroxidase (Sigma Aldrich, USA) by sodium periodate method [13] to use as detection antibody. HRP-conjugated OmpW IgY was diluted to 1 : 1000, 1 : 2000, 1 : 4000, 1 : 8000, 1 : 16,000, 1 : 32,000 and 1 : 64,000 in 1% BSA in PBS buffer. Checkerboard titration was performed to determine the optimal amounts of detection and capture *α*-OmpW antibodies. The positive and negative controls were tested in triplicate, and the mean OD of them was used for evaluation.

For *α*-OmpW ELISA, the microplates (Nunc, Denmark) were coated with 100 *µ*l of purified *α*-OmpW antibodies (1.25 *µ*g/mL) at 4°C, overnight. After washing twice with PBS-T, the microplates were blocked for 1 hour at room temperature with PBS containing 1% BSA. Then, 100 microliter of each bacterial suspension was added to each well and incubated at 37°C for 1 hour. The microplates were washed four times with PBS-T, and 100 *µ*l of 1 : 16,000–diluted HRP-conjugated *α*-OmpW antibodies was added to wells. After 1-hour incubation at 37°C and four times of washing, 100 *µ*l of TMB was added to each well and the plates were incubated at room temperature, avoiding direct light. The enzymatic reaction was stopped after 15 minutes by adding 50 *µ*l of 1 M HCl. The optical density of wells was read at 450 nm. The mean OD of two blank wells was subtracted from the OD value of sample wells.

### 2.8. *α*-CtxB ELISA for Detection of Cholera Toxin

In order to optimize *α*-CtxB ELISA, the bacterial supernatants of *V. cholera* (PTCC No.: 1611) and *E. coli* (PTCC No.: 1395) were cultured under AKI-SW condition to stimulate cytotoxin production [14], and the supernatant of *V. cholerae* and *E. coli* culture media were used as positive and negative controls, respectively. For the development of *α*-CtxB ELISA, a part of dialyzed *α*-CtxB antibodies was diluted to 10, 5, 2.5, 1.25, 0.6, 0.3, and 0.15 *μ*g/mL in coating buffer. Then, the biotin-conjugated monoclonal antibody against *V. cholerae* toxin B subunit (Novus Biologicals, USA) was diluted to 1 : 1000, 1 : 2000, 1 : 4000, 1 : 8000, 1 : 16,000, 1 : 32,000 and 1 : 64,000 in 1% BSA in PBS buffer. Checkerboard titration was performed to determine the optimal amounts of detection and capture *α*-CtxB antibodies. The positive and negative controls were tested in triplicate, and the mean OD of them was used for evaluation.

For *α*-CtxB ELISA, the microplates (Nunc, Denmark) were coated with 100 *µ*l of purified *α*-CtxB antibodies (0.6 *µ*g/mL) at 4°C, overnight. After washing twice with PBS-T, the microplates were blocked for 1 hour at room temperature with PBS containing 1% BSA. Then, 100 microliters of each sample was added to corresponding wells and incubated at 37°C for 1 hour. The microplates were washed four times with PBS-T, and 100 *µ*l of 1 : 32,000 biotin-conjugated anti-*V. cholerae* toxin B subunit monoclonal antibody (Novus Biologicals, USA) was added to wells and incubated at 37°C for 1 hour. After 1-hour incubation at 37°C and 4 times washing, 1 : 5000 dilution of HRP-conjugated streptavidin (Sigma Aldrich, USA) was added to each well and incubated for 1 hour at 37°C. Then, the plates were washed for 4 times, 100 *µ*l of TMB was added to each well, and the plates were incubated at room temperature avoiding direct light. The enzymatic reaction was stopped after 15 minutes by adding 50 *µ*l of 1 M HCl. The optical density of wells was read at 450 nm. The mean OD of the two blank wells was subtracted from the OD value of sample wells.

### 2.9. Determination of the Analytical Sensitivity of the Developed Assays

The sensitivity of the developed ELISAs was determined by testing tenfold serial dilutions of 0.5 McFarland equivalence suspension of *V. cholerae* O1 culture ranging from 1.5 × 10^8^ to 1.5 × 10^1^ colony-forming units/mL (for *α*-OmpW ELISA) and twofold serial dilutions of recombinant CtxB ranging from 1000 pg/mL to 7.8 pg/mL (for *α*-CtxB ELISA). Then, the *α*-OmpW and *α*-CtxB ELISAs were run as described in Sections 2.7 and 2.8, respectively. In each plate, 20 wells were considered as blank and 100 *µ*l of dilution buffer (1% BSA in PBS) was added to blank wells instead of antigen. The optical density of each well was read at 450 nm. The mean and standard deviation of 20 blank wells were determined. Then, the LoB (Limit of Blank) and the absorbance of LoD (Limit of Detection) were calculated for each assay using the following formulas [15, 16]:

(1) $$\begin{array}{c} \text{LOB}={\text{Mean}}_{\text{Blank}}+1.645\left({\text{SD}}_{\text{Blank}}\right),\\ {\text{Ab}}_{\text{LOD}}={\text{Mean}}_{\text{Blank}}+3\left({\text{SD}}_{\text{Blank}}\right). \end{array}$$

The standard curve and fitted equation of serial dilutions were used to determine the limit of detection of two assays.

### 2.10. Determination of the Analytical Specificity of the Developed Assays

The specificity of the two assays was determined by testing the supernatant of different bacterial strain (Table 1) cultures with concentration equivalent to 1 McFarland by *α*-OmpW and *α*-CtxB ELISAs, as described above.

*E. coli* (ETEC) (PTCC: 1399) was used for further evaluation of possible cross-reactivity of heat-labile (LT) toxin of ETEC and *α*-CtxB ELISA. The method suggested by Letícia B. Rocha et al. using lincomycin and triton X-100 was performed for production and release of LT toxin [17], and the supernatant of the culture media was tested by *α*-CtxB ELISA.

### 2.11. Evaluation of the Analytical Sensitivity and Specificity of the Assays in Spiked Stool Matrix Sample

Five different formed human stool samples were used as matrices for spiking with *V. cholerae* (PTCC No.: 1611) and recombinant CtxB. These samples had been tested negative for enteropathogenic bacteria (*Shigella* spp., *Salmonella* spp., *Aeromonas* sp., *Plesiomonas* sp., and *Yersinia enterocolitica*) by routine culture. After that, the samples were pooled to use as stool matrix for further processing.

A bacterial suspension of *V. cholerae* (PTCC No.: 1611) with the density equivalent to 0.5 McFarland was prepared in PBS, then tenfold serial dilutions, ranging from 1.5 × 10^8^ to 1.5 × 10^2^ cfu/mL, were prepared from this suspension. A stock solution of recombinant CtxB with the final concentration of 1000 pg/ml was prepared in PBS, and twofold serial dilutions, ranging up to 15.6 pg/ml, were prepared from the stock solution.

Five grams of the pooled stool matrix was diluted with PBS to a final volume of 30 mL. 500 *μ*L of the diluted stool matrix was then combined with the same volume of the prepared CtxB and bacterial suspensions to create the following rice-water-stool–like consistency samples:

- Seven serial dilutions containing 7.5 × 10^7^ to 7.5 × 10^1^ cfu/mL of *V. cholerae*
- Seven serial dilutions containing 500 to 7.8 pg/mL of recombinant CtxB

All the prepared spiked stool specimens and a sample without spiking, as negative control, were extracted by the following procedure; 500 *μ*L of each sample was mixed thoroughly with 500 *μ*L of extraction buffer, including 2% BSA in PBS and 0.02% sodium azide as a preservative. After vortexing, the samples were centrifuged at 3000×*g* for 5 minutes, and the supernatants were transferred into fresh microtubes. The cleared samples were analyzed in duplicate by *α*-OmpW and *α*-CtxB ELISAs, as described in Sections 2.7 and 2.8, respectively. The average optical density of each sample was used for the evaluation of the sensitivity and specificity of the two ELISA methods in spiked stool matrix samples. For this purpose, the samples with an optical density (OD) greater than the limit of detection of two assays, calculated in Section 2.9, were defined as positive.

### 2.12. Statistical Analysis

All the statistical analyses were performed using SPSS 16.0 statistical software. Significant differences between means of ELISA signals for the determination of specific IgY antibodies were detected by *t*-test. The means of ELISA signals between weeks after immunization were compared by repeated measure ANOVA. *P* value <0.05 was considered as statistically significant.

## 3. Results

### 3.1. Amplification and Cloning of *ompW* and *ctxB* Genes

Electrophoresed and ethidium bromide-stained PCR products of both genes showed the expected sizes of 651 and 372 bp for *ompW* and *ctxB* genes, respectively. Transformation of ligation products into *E. coli* leads to the growth of kanamycin-resistant colonies, indicating the presence of recombinant vectors in BL21 (DE3) bacteria. Colony PCR and enzymatic digestion of recombinant vectors confirmed correct ligation of inserts into vectors.

### 3.2. Expression and Purification of Recombinant OmpW and CtxB Proteins

SDS-PAGE analysis of IPTG-induced bacteria revealed the expected 27 kDa band for OmpW and 17 kDa band for CtxB protein. The final yield of purified recombinant OmpW and CtxB was 59 and 38 mg per one liter of bacterial culture, respectively.

### 3.3. Immunization and IgY Purification

After immunization, no decline in egg-laying capacity was seen (*P* value <0.05) and the immunized chickens still laid an average of one egg per day, showing no adverse effects or laying decrease caused from immunization. Extraction and purification of specific antibodies were successfully carried out by PEG 6000. The average yield (±SD) of purified antibodies was 66.5 ± 1.80 mg *α*-OmpW and 50.9 ± 2.23 mg *α*-CtxB per egg.

### 3.4. Evaluation of Purified IgY Antibodies

**Figure 1:**
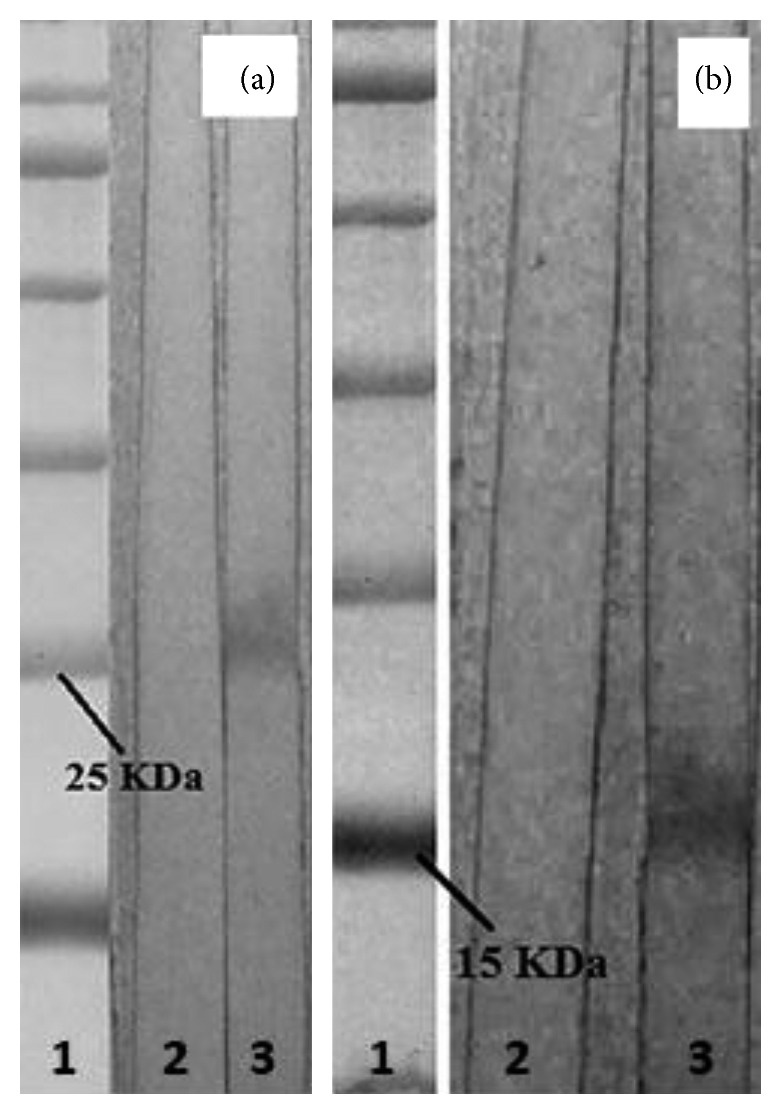
Binding capability evaluation of *α*-OmpW and *α*-CtxB antibodies. (a) Strip 1: protein size marker; Strip 2: purified IgY from control group chicken showed no band with recombinant OmpW; and Strip 3: the band of 27 kDa recombinant OmpW reacted with *α*-OmpW antibodies. (b) Strip 1: protein size marker; Strip 2: purified IgY from control group chicken showed no band with recombinant CtxB; and Strip 3: the band of 17 kDa recombinant CtxB reacted with *α*-CtxB antibodies.

**Figure 2:**
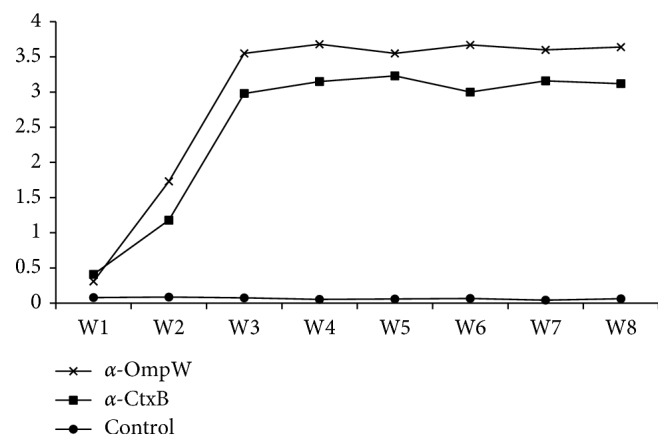
Changes in the specific IgY levels in the egg yolk after the last immunization in immunized and control groups.

Binding capability of the purified antibodies was evaluated by western blotting. The antibodies bound specifically to their target proteins producing 27 and 17 kDa bands for *α*-OmpW and *α*-CtxB, respectively. No band was appeared by purified IgY from control group chicken (Figure 1). The activity of purified antibodies was measured from indirect ELISA absorbance data. The results of the purified IgY antibodies obtained from immunized chickens showed specific reaction to recombinant proteins. No specific antibodies against OmpW and CtxB antigens were seen in the control group (*P* value <0.0001). Indirect ELISA assays indicated specific binding of *α*-OmpW and *α*-CtxB antibodies to the corresponding targets as expected. The production rate of specific *α*-OmpW and *α*-CtxB during the egg collection time is shown in Figure 2. According to the results, the antibody levels started to increase significantly two weeks after the final immunization (*P* value <0.05). The antibody titer peaked in the third week and remained at a relatively constant level until the end of egg collection (eighth week).

### 3.5. Optimization of Two Sandwich ELISAs

**Figure 3:**
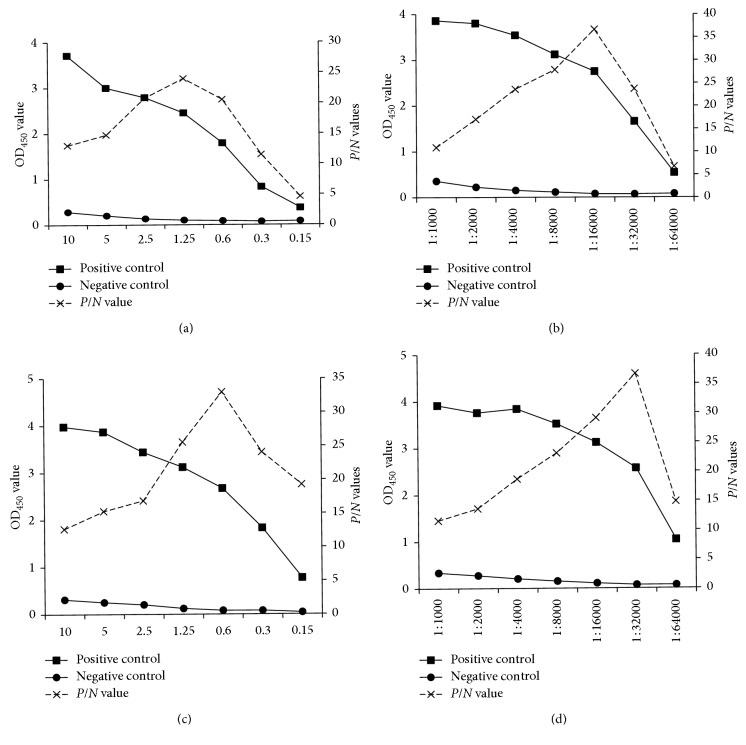
Optimization of *α*-OmpW and *α*-CtxB ELISAs. (a) Coating concentration of *α*-OmpW and (b) HRP-labeled *α*-OmpW dilutions. (c) Coating concentration of *α*-CtxB and (d) biotin-*α*-CtxB dilutions.

The sandwich ELISAs were optimized using the checkerboard method. According to the OD450 and positive/negative values, the ideal concentration of capture antibodies for coating were set at 1.25 and 0.6 *μ*g/mL for *α*-OmpW and *α*-CtxB ELISAs, respectively (Figures 3(a) and 3(c)). The optimal dilution of HRP-*α*-OmpW and biotin-conjugated anti-*V. cholerae* toxin B subunit antibodies was 1 : 16,000 and 1 : 32,000, respectively (Figures 3(b) and 3(d)).

### 3.6. Analytical Sensitivity and Specificity of the Developed Assays

Analytical sensitivity of the newly developed assays was determined by serial dilutions of *V. cholerae* and recombinant CtxB to construct a standard curve. The resulted equations were used to calculate the limit of detections.  Table 3 shows the final results of the sensitivity evaluation. In addition, testing different bacterial strains (Table 1) with *α*-OmpW and *α*-CtxB ELISA did not show signals higher than their limit of blanks (OD: 0.254 and OD: 0.123, respectively), indicating no cross-reactivity with the tested bacteria. Testing the supernatant of *E. coli* (ETEC) culture grown under LT-toxin stimulation condition by *α*-CtxB ELISA resulted in the mean optical density of 0.29, which was higher than the absorbance of LoD of the assay.

### 3.7. Analytical Sensitivity and Specificity of the Assays in Spiked Stool Matrix Sample

In *α*-OmpW, the spiked sample contained at least 7.5 × 10^3^ cfu/mL of *Vibrio chloerae* showed an optical density (OD) greater than the limit of detection of the assay, i.e., 0.373. In CtxB ELISA, the optical density of the sample spiked with 62.5 pg/mL of recombinant CtxB was higher than the limit of detection of the assay, i.e., 0.153. In both assays, the final concentrations of the spiked samples are calculated by considering the dilution factor used for preparation of cleared sample. The results are shown in Table 4.

## 4. Discussion

Because of its rapid spread and vast epidemics, cholera associates with a heavy economic burden in the involved endemic area. According to the Global Health Observatory (GHO) data, in 2016, 132,121 cholera cases and 2420 deaths were reported to WHO worldwide [18]. Dealing with this threat necessitates paying more attention through improving global health. Vaccination and employing rapid diagnostic methods are two major solutions to achieve this goal. Early diagnosis of cholera outbreak leads to the prevention, control, and management of the disease. As a result, employing rapid, simple, and inexpensive diagnostic methods is considered as a very critical tool to control the spread of cholera [19].

Considering the need for a rapid diagnostic method, in this study, the avian antibodies targeting *V. cholerae* antigens have been produced to be used for the development of two sandwich ELISA methods, *α*-OmpW and *α*-CtxB ELISA, to be potentially used for the specific and sensitive detection of *V. cholerae* and its cytotoxin.

For this purpose, a specific polyclonal antibodies–targeting outer membrane protein W (OmpW) has been employed for the detection of *V. cholerae*. A comprehensive study on *ompW* gene conducted by Nandi et al. demonstrated that its coding sequence is highly conserved between *V. cholerae* strains and can be used for diagnostic purposes [10]. In addition, immunogenicity evaluation shows that OmpW is a strong immunogenic protein [20]. These two features make this antigen an ideal target to be used in diagnostic immunoassays. In addition, for discriminating toxigenic and nontoxigenic strains, subunit B of cholera toxin (CtxB) was used. Cholera toxin is produced by the CTXф residing within the bacterial chromosome. The sequence of cytotoxin gene of CTXф is conserved in classic and El Tor strains, including some single-nucleotide polymorphisms [21]. In addition, there are some studies proving the suitability of CtxB as a specific marker for discrimination of toxigenic strains [4, 22].

Here, two avian polyclonal antibodies were raised against recombinant OmpW and CtxB antigens of *V. cholerae*. To the best of our knowledge, current study is the first report on using IgY in the detection of toxigenic *V. cholerae*. Avian egg antibodies are a very rich and cost-effective source of these valuable molecules. Noninvasive sampling, high antibody amount (50–100 mg/egg), no interference with mammalian immunoglobulins (rheumatoid factors), HAMA (human anti-mouse antibody), and complement are the most important functional characteristics of avian antibodies [23]. Dose and molecular weight of antigen, the type of adjuvant, the route of administration, and the chicken age, breed, and keeping conditions all influence the immune response of the immunized chicken [24]. In the current study, we used the intramuscular injection of antigen formulated with Freund's adjuvant. Production of the specific antibodies started to increase two weeks after immunization (*P* value <0.05), peaked at the third week, and remained in constant level until the end of study (eighth week).

In this study, the egg yolk antibodies were extracted and purified by polyethylene glycol method [12]. There are different researches on IgY purification, reporting different yields of purified IgY. Pauly et al. reported the purification yields ranging from 28.8 to 60 mg per egg yolk for different specific antibodies [12, 25]. In another study for the production of IgY against soluble antigens of *Toxoplasma gondii*, the researchers were able to purify 48 mg of antibody per egg [26]. According to their report, Wen et al. yielded 76.5 mg of IgY antibody against inactivated influenza B virus [27]. Here, the average yield of purified *α*-OmpW and *α*-CtxB is 66.5 and 50.9 mg per egg, respectively. The yield obtained in the present study is acceptable and comparable with the other similar experiments.

The purified specific IgY antibodies were used for the development of the two sandwich ELISAs. Performing checkerboard titration allowed a simple and quick optimization of the involved factors. Using purified IgY and applying precise optimization procedure led to development of the specific ELISAs with low detection limits, which have a significant advantage over the existing methods. Currently, various methods for the detection of *V. cholerae* have been introduced. In 2003, Pasteur Institute of Paris developed an immunochromatographic method based on anti-lipopolysaccharide antibodies. The detection limit of the assay was 10^7^ cfu·mL^−1^ of O1 strain and 10^6^ cfu·mL^−1^ of O139 [9]. In another research, Tuteja et al. designed a dipstick sandwich ELISA to detect OmpW and CtxB antigens. The analytical sensitivity of the developed assay was 10^4^ cfu·mL^−1^ for OmpW and 60 pg/mL of CtxB [22]. Also in the immunochromatographic test strip designed by Yamasaki et al., the reported limit of detection was 10 ng/mL of cholera toxin [28]. In the current research, the limit of detection of OmpW is less than 1000 cfu/mL and that of CtxB is around 33 pg/mL. Therefore, our newly developed assays show higher sensitivity than other similar works. The yolks of immunized chickens are a very rich source of IgY, which leads to obtaining high-purity antibodies and consequent high sensitivity of the developed immunoassays. Evaluation of analytical specificity by different bacterial strains (Table 1) with *α*-OmpW and *α*-CtxB ELISA did not show any cross-reactivity, but as the heat-labile toxin (LT) of enterotoxigenic *E. coli* (ETEC) shares around 80 percent homology with *V. cholerae* CT [29], we further evaluated nonspecific reaction of *α*-CtxB ELISA in the supernatant of enterotoxigenic *E. coli* cultured under LT-toxin stimulation condition. The result of *α*-CtxB ELISA in this condition shows cross-reactivity with *V. cholerae* cytotoxin. Increasing the LoD of *α*-CtxB ELISA from 0.153 to a value above 0.29 would be helpful to overcome the problem resulting from the nonspecific reaction with ETEC enterotoxin. Considering the value of 0.30 for the LoD of *α*-CtxB ELISA results in the limit of detection to be around 80 pg/mL, which still shows an acceptable sensitivity for this test. On the other hand, as these ELISAs are considered to be used as two subsequent tests, only the specimens which contain *V. cholerae* (OmpW-positive samples) will be tested by *α*-CtxB ELISA. This decreases the probability of cross reaction by ETEC enterotoxin.

The evaluation of the analytical sensitivity of the assays using spiked stool samples shows that the stool matrix does not lead to false-positive results in negative control, and their sensitivity are at least 7500 cfu/mL and 62.5 pg/mL for *α*-OmpW and *α*-CtxB ELISAs, respectively. The sensitivity of the assays using fecal sample decreased in comparison with the analytical sensitivity obtained by antigenic and bacterial suspensions in PBS, i.e., 1000 cfu/mL and 33 pg/mL for OmpW and CtxB, respectively. The main factor causing the decrease in sensitivity is the need to dilute stool specimen for preparing clear samples, which reduces the sensitivity of the assays by at least two times. As usual, pathogen load for *V. cholerae* in rice-water stool are between 10^10^ and 10^12^ colony-forming units per liter [30], even with this decreased sensitivity, *α*-OmpW ELISA is still efficient for the detection of *V. cholerae* in infected individuals. In addition, this method can be performed on fecal samples cultured in alkaline peptone water for 4 hours. Using 4-h-enriched fecal samples will significantly increase the sensitivity [6] and, in addition, it will eliminate the need for making clear samples. Furthermore, to use this method for environmental samples, there is no need for making clear samples, and thus, the sensitivity of the method will not be reduced.

High analytical sensitivity and specificity of the developed assays, proved in this study, show the potential usefulness of them to be applied for diagnostic purposes. Further evaluation of the assays requires clinical sensitivity and specificity in a large-field trial using a large number of human stool and environmental samples. We plan to determine these parameters in *α*-OmpW and *α*-CtxB ELISAs in the future.

## 5. Conclusions

Due to the unique biological characteristics, IgY antibodies promise to resolve some of the current technical issues in immunological assays. Based on these features, we produced specific IgY antibodies for the development of two IgY-based sandwich ELISAs for the specific and sensitive detection and discrimination of toxigenic *V. cholerae*. As the specificity of the designed assays is 100% and their sensitivities are higher than current rapid tests, they can be used as a reliable and robust tool to be potentially used for the detection of toxigenic *V. cholerae* in clinical and environmental samples. To the best of our knowledge, this study is the first report on using IgY in detection of *V. cholerae*.

## Figures and Tables

**Table 1:** Bacterial strains used to evaluate the specificity of *α*-OmpW ELISA.

Strain	Number
*Acinetobacter baumannii*	PTCC: 1797
*Aeromonas hydrophila*	MCI: 1096
*Bacillus cereus*	PTCC: 1154
*Bacteroides fragilis*	MCI:1011
*Enterobacter aerogenes*	PTCC: 1221
*Enterococcus faecalis*	PTCC: 1237
*Escherichia coli*	PTCC: 1395
*Escherichia coli EPEC(M)O55:K 59*	PTCC: 1269
*Klebsiella oxytoca*	PTCC: 1402
*Lactobacillus acidophilus*	PTCC: 1643
*Proteus mirabilis*	PTCC: 1776
*Pseudomonas aeroginosa*	PTCC: 1310
*Salmonella enterica*	PTCC: 1709
*Shigella dysenteriae*	PTCC: 1188
*Yersinia enterocolitica*	PTCC: 1151
*Escherichia coli (ETEC)*	PTCC: 1399

**Table 2:** The primer sets used for amplification of *ompW* and *ctxB* genes.

*ompW*	Forward: aaaggatccATGAAACAAACCATTTGC
	Reverse: tctctcgagTTAGAACTTATAACCACCC

*ctxB*	Forward: aaaggatccATTAAATTAAAATTTGGTG
	Reverse: aatctcgagTTAATTTGCCATACTAATTG

Underlined nucleotides are the restriction sites of *Bam*HI and *Xho*I.

**Table 3:** The result of analytical sensitivity calculation for newly developed ELISAs.

ELISA	Mean OD of blanks	SD of blanks	LOB	Absorbance of LoD	Line equation	LoD
*α*-OmpW	0.110	0.087	0.254	0.373	*y* = 0.1965*x* − 0.2103	933 cfu·mL^−1^
*α*-CtxB	0.087	0.022	0.123	0.153	*y* = 0.0031*x* + 0.0508	33 pg·mL^−1^

**Table 4:** The result of analytical sensitivity and specificity of the developed ELISAs in spiked stool sample.

*α*-OmpW			*α*-CtxB		
Spiked conc. (Cfu·mL^−1^)	Final conc.^*∗*^ (cfu·mL^−1^)	OD	Spiked conc. (pg·mL^−1^)	Final conc.^*∗*^ (pg·mL^−1^)	OD
7.5 × 10^7^	3.8 × 10^7^	2.86	500	250	1.73
7.5 × 10^6^	3.8 × 10^6^	1.92	250	125	1.02
7.5 × 10^5^	3.8 × 10^5^	1.25	125	62.5	0.45
7.5 × 10^4^	3.8 × 10^4^	0.69	**62.5**	**31.3**	**0.21**
**7.5 × 10** ^**3**^	**3.8 × 10** ^**3**^	**0.41**	31.3	15.6	0.14
7.5 × 10^2^	3.8 × 10^2^	0.18	15.6	7.8	0.08
7.5 × 10^1^	3.8 × 10^1^	0.07	7.8	3.9	0.06
0 (negative control)	0	0.08	0 (negative control)	0	0.06

^*∗*^Final concentration is calculated by considering the dilution factor used for preparation of cleared sample.

## Data Availability

The data used to support the findings of this study are available from the corresponding author upon request.
