# Corrigendum to “Development of IgY-Based Sandwich ELISA as a Robust Tool for Rapid Detection and Discrimination of Toxigenic *Vibrio cholera*”

**DOI:** 10.1155/2019/4164982

**Published:** 2019-03-04

**Authors:** Mahdiye Bayat, Alireza Khabiri, Behzad Hemati

**Affiliations:** ^1^Department of Microbiology, Islamic Azad University-Karaj Branch, Karaj 3148635731, Iran; ^2^Diagnostic Biotechnology Unit, Pasteur Institute of Iran, Research and Production Complex, Karaj, Iran

In the article titled “Development of IgY-Based Sandwich ELISA as a Robust Tool for Rapid Detection and Discrimination of Toxigenic *Vibrio cholera*” [1], the affiliation of the first author should be “Department of Microbiology, Islamic Azad University-Karaj Branch, Karaj 3148635731, Iran” only. The updated authors' list and affiliations are shown above.

## References

[1] Bayat M., Khabiri A., Hemati B. (2018). Development of IgY-based sandwich ELISA as a robust tool for rapid detection and discrimination of toxigenic *Vibrio cholerae*. *Canadian Journal of Infectious Diseases and Medical Microbiology*.

